# Effect of glycerin on the physical properties of polyvinyl alcohol/sodium alginate blend

**DOI:** 10.1038/s41598-024-75235-1

**Published:** 2024-10-23

**Authors:** Ahmed Fahmy, Rania Badry, Rasha M. Khafagy, Medhat A. Ibrahim

**Affiliations:** 1 Physics Department, Faculty of Women for Arts, Science and Education, Ain Shams University, Cairo, 11757 Egypt; 2 Spectroscopy Department, National Research Centre, 33 El-Bohouth St., Dokki, Giza, 12622 Egypt; 3 Molecular Modeling and Spectroscopy Laboratory, Centre of Excellence for Advanced Science, National Research Centre, 33 El-Bohouth St., Dokki, Giza, 12622 Egypt

**Keywords:** Sodium-ion batteries, PVA, Na Alg, DFT, MESP, QSAR descriptors and thermal parameters, Materials science, Physics

## Abstract

Because of the abundance of sodium resources, sodium-ion batteries (NIBs) offer a promising alternative electrochemical energy storage solution. One of the current roadblocks to the development of NIBs technology is a lack of electrode materials capable of reversibly storing/releasing sodium ions for a sufficiently long time. Thus, this work aims to study, theoretically, the effect of glycerin incorporation on polyvinyl alcohol (PVA)/sodium alginate (Na Alg) blend as electrode materials for NIBs. The electronic, thermal, and quantitative structure-activity relationship (QSAR) descriptors of polymer electrolytes based on a blend of PVA and Na Alg and glycerin are the main topics of this work. These properties are examined here using semi-empirical methods and the density functional theory (DFT). Bandgap energy (E_g_) is examined because the structural analysis reveals details regarding the interactions between PVA/Na Alg and glycerin. The findings indicate that the addition of glycerin caused the E_g_ value to drop to 0.2814 eV. The molecular electrostatic potential surface, or MESP, shows the electron-rich and deficit regions throughout the electrolyte system as well as the distribution of molecular charges. Thermal parameters that are studied include enthalpy (H), entropy (ΔS), heat capacity (Cp), Gibbs’ free energy (G), and heat of formation. Additionally, the study examines several QSAR descriptors, such as total dipole moment (TDM), total energy (E), ionization potential (IP), Log P, and Polarizability. The results show that H, ΔS, Cp, G, and TDM increased with increasing temperature and glycerin content. Meanwhile, heat of formation, IP, and E decreased, improving reactivity and polarizability. Additionally, the cell voltage increased to 2.488 V due to glycerin addition. The overall DFT and PM6 calculations of cost-effective PVA/Na Alg based glycerin electrolytes indicate that they can partially replace lithium-ion batteries due to their multifunctionality, but requires further improvement and investigations.

## Introduction

Despite their widespread use, Li-ion batteries (LIBs) face limitations due to their short lifespan, high cost, and safety concerns. Na-ion batteries (NIBs) may be a viable alternative to LIBs due to the widespread availability, low cost, and nontoxicity of Na. Rechargeable batteries (RBs) used in electrochemical devices are becoming an increasingly important energy storage system^1^. A salt-ion battery relies heavily on its electrolyte, which facilitates the movement of ions and generates current^2,3^. Liquid electrolytes primarily consist of metallic salt and an organic solvent. Practical applications require careful consideration of liquid electrolyte safety, especially when batteries are subjected to thermal or electrical abuse^4^.

Na-ion batteries (NIBs) have the potential to replace LIBs in the near future due to their abundance in the sea, nontoxicity of sodium, and low material cost^5^. Synthesis of nanomaterials has accelerated the development of storage, electronic, and optical devices^6^. Numerous publications demonstrate the use of various nanostructures, such as metal oxides, graphene, nanotubes, and fullerenes, in ion batteries^7–10^. Research has focused on developing suitable anode materials for NIBs, including polymers, because of their multifunctionality and environmental friendliness. Research interest in the development of rechargeable polymer batteries will undoubtedly increase. It’s highly likely that newly developed polymer electrode materials with unique structures and characteristics will open the door to the creation of environmentally friendly energy storage technologies^11^. Although several polymer electrode materials for sodium-ion batteries have been studied, the field is still in its early stages of development. For the purpose of sodium-ion batteries, more polymeric materials with various structural configurations must be investigated. Based on what we currently know about the mechanisms underlying the storage of sodium ions in polymer electrode materials, carbonyl groups, radicals, and heteroatoms in conjugated systems may serve as the active sites for interactions with sodium ions. Consequently, designing new polymers with a high density of these active sites is highly desirable. Gel polymer electrolytes (GPE) are an alternate technique that improves high reliability, ionic conductivity, no leakage, high flexibility, and good performance^12^.

Polymer matrixes include materials such as PVA and polyethylene oxide (PEO)^13^. GPEs immobilize the liquid electrolyte in a polymeric matrix, reducing the risk of leakage compared to commercial separators^14^. PVA is a synthetic, biodegradable polymer. It has a high dielectric constant and is inexpensive and non-toxic. This material is known for its ability to form films, maintain chemical stability, and retain sticky properties. It also has functional (OH) groups and a high-density cross-linking potential^15–17^. To improve the conductivity of PVA-based polymer electrolytes, polymer blending, plasticizers, composite addition, and in-situ polymerization techniques have been used to reduce matrix crystallinity and increase chain flexibility^18–20^.

Blending is an important method of developing polymeric materials used in industry. The polymer blends are commonly used to (1) improve the processability of a natural polymer for industrial applications; (2) improve the chemical, physical, and mechanical properties of a biodegradable material; and (3) adapt to frequent changes in demand for new materials in the food-packaging industry. Polymer blends, as opposed to copolymerization, are a low-cost process that uses simple physical processes rather than complicated chemical processes to achieve the desired properties^21^. To form homopolymeric materials, different polymers can interact through dipole-dipole forces, hydrogen bonds, or the formation of charge transfer complexes^22,23^. Blend materials made from natural polymers and synthetic polymers can combine the benefits of good biocompatibility and excellent mechanical properties to create a type of prominent material at a low production cost^24,25^. Therefore, there has been a lot of interest in the creation of biologically significant polymeric materials through the blending of synthetic and natural polymers. PVA can be combined with sodium alginate (Na Alg), cellulose, chitosan, and starch^26^.

Na Alg, a natural polymer, is an anion-based polysaccharide obtained from marine brown algae. Na Alg is made up of β-(1–4)-linked D-mannuronate (M) and α-(1–4)-linked L-guluronate (G), arranged in a homopolymeric form (poly-M and poly-G) with heteropolymeric blocks (MG or GM)^27^. The contents of M and G blocks, as well as their relative proportions, have a significant impact on alginate’s chemical and physical properties^28,29^. Na Alg has been extensively used and researched due to its biodegradability, biocompatibility, low cost, good film-forming, and non-toxicity properties. However, the abundance of free hydroxyl (OH) and carboxylate (COO) along the alginate backbone contributes to alginate’s high hydrophilicity. However, alginate has poor mechanical properties due to its brittleness and rigidity. Thus, alginate is combined with other synthetic materials to improve water sensitivity and mechanical properties^30,31^.

The DFT computation is frequently used to assess the feasibility of new material preparation prior to designing new electrode materials. Additionally, to confirm and predict experimental results, save time, reduce chemical waste, and predict the behavior of interactions, scientists use molecular modeling^32^. Molecular modeling has become a powerful and important branch of science in several fields, such as material science, nanomaterials, computational chemistry, and drug design^33,34^. Using modeling programs, scientists could directly get molecular data, including energies (heat of formation, ionization potential, activation energy, etc.) and geometries (bond angles, bond lengths, and torsion angles)^35^. Also, electronic properties (charges, HOMO and LUMO band gap energy, electron affinity), spectroscopic properties (characteristic vibrational modes and intensities such as FTIR spectra), and bulk properties (volumes, diffusion, viscosity, modulus, etc.) can be calculated^36^.

Due to its high energy density, LiNiPO_4_ (operating at approximately 5.1 V) exhibits potential advantages in the competition of cathode materials for LIBs. To fully utilize the high-voltage benefits of LiNiPO_4_, the operating voltage must be lowered because the high-voltage electrolyte that was developed can only stay relatively stable in the range of less than 4.8 V. Zhang et al. studied the effect of all 3d, 4d, and 5d transition metals doped into the Ni site of LiNiPO_4_ to screen out the doped models with excellent electrochemical performance and regulate the operating voltage of LiNiPO_4_ while maintaining the relative stability of its electrochemical properties. The lowest operating voltages they obtained are 4.21, 3.76, and 3.50 for LiNiPO_4_ doped with Ti, Nb, and Ta, respectively^37^.

Accordingly, this work aims to study the effect of glycerin as a plasticizer on the electronic, QSAR descriptors, and thermal properties of PVA/Na Alg theoretically utilizing quantum mechanical calculations for possible application as rechargeable ion batteries. The molecular interactions between the PVA/Na Alg models and glycerin were analyzed using Bader’s quantum theory of atoms in molecules (QTAIM).

## Calculation details

Model molecules representing PVA interacted with Na Alg and then with glycerin were subjected to optimization utilizing DFT. Models were subjected to calculations using Gaussian 09 software^38^ at the Spectroscopy Department, National Research Centre, Cairo, Egypt. Models were optimized with the DFT at the B3LYP/6-311G(d, p) level^39–42^. To validate the interaction between the studied models, a frequency study conducted at the same theoretical level demonstrated the stability of optimized geometries. The absence of negative frequencies across all estimated frequencies highlights the supposed structures at real positive and true minimums in the potential energy surface. Physical parameters such as TDM, HOMO/LUMO band gap energy, and MESP were computed at the same quantum mechanical level of theory. Additionally, some thermal parameters were calculated, such as the final heat of formation, free energy, entropy, enthalpy, and heat capacity, using the equations presented in Table 1. The Quantum Theory of Atoms in Molecules (QTAIM) analysis was conducted for the studied models to point out the interactions that took place on the surface of the studied structures. These computations were performed with the “output = wfn” command in the Gaussian 09 softcode, then visualized with the Avogadro softcode^43^.

**Table 1:** Mathematical equations representing some thermal parameters and QSAR descriptors.

Property	Equation
1. Enthalpy (H)	H = E + PV (1)
2. Entropy (ΔS)	ΔS = ΔQ T (2)
3. Heat capacity (C_p_)	C_p_ = ΔH/ΔT (3)
4. Free energy (ΔG)	ΔG = ΔH–TΔS (4)
5. Ionization potential (IP)	IP = a + bq + cq (5)
6. Log (P)	Log (P) = Concentration in Organic/Concentration in Aqueous (6)

where E is the internal energy, P is the pressure, V is the volume, and Q is the change in the heat between a system and its surroundings, T is the temperature, ΔH is the change in enthalpy, ΔG is the change in free energy, ΔS is the change in entropy, a and b are the vibrational parameters, q is the charge of an atom, and C is the electron density of an atom^44,45^. Finally, the same structures were optimized, and the QSAR parameters were carried out at the PM6 level using the SCIGRESS soft code^46^ at the Spectroscopy Department, National Research Centre, Cairo, Egypt.

## Results and discussion

### Building molecular models

**Fig. 1:**
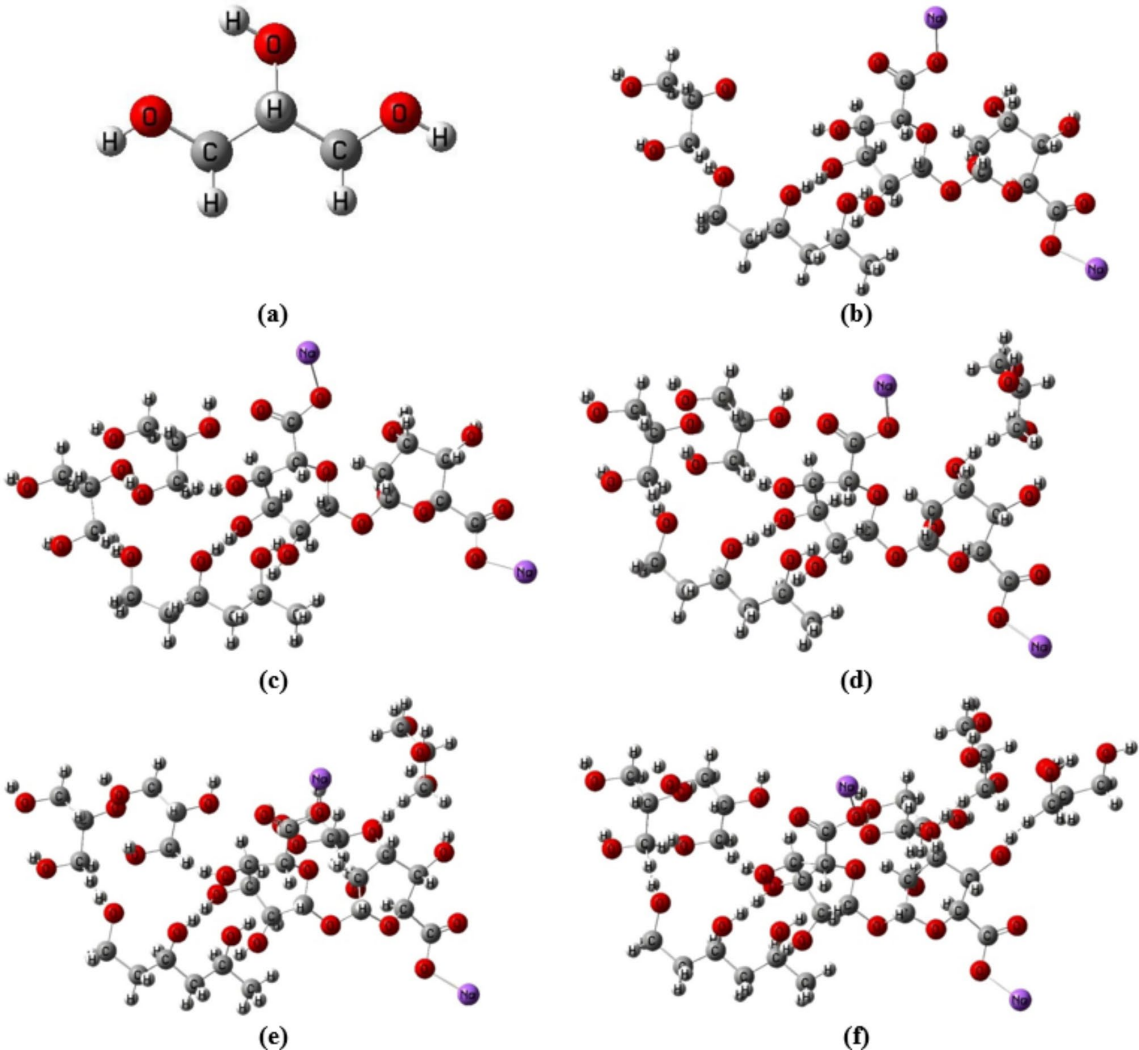
Optimized structures of: (**a**) Gly and 3PVA − 2Na Alg interacted with (**b**) 1 Gly, (**c**) 2 Gly, (**d**) 3 Gly, (**e**) 4 Gly and (**f**) 5 Gly.

**Fig. 2:**
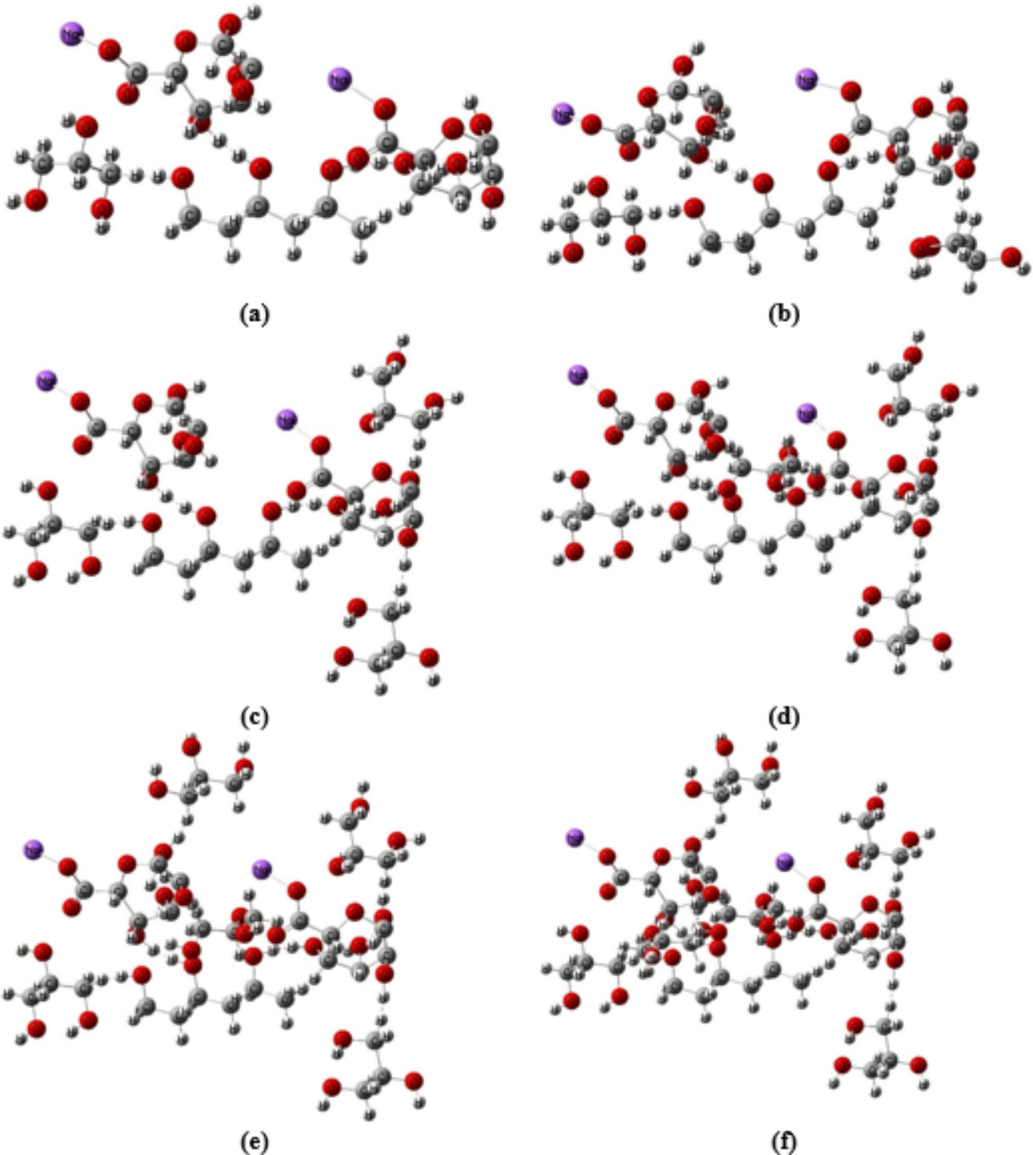
Optimized structures of Term 1Na Alg- 3PVA –Mid 1Na Alg interacted with (**a**) 1 Gly, (**b**) 2 Gly, (**c**) 3 Gly, (**d**) 4 Gly, (**e**) 5 Gly, and (**f**) 6 Gly.

Glycerin is used as a plasticizer in the most likely model describing how three units of PVA interact with two units of Na Alg, which was estimated in our previous work^47^. As previously indicated, there are two possibilities for PVA and Na Alg interaction. In comparison to other structures under consideration, the two models specified as 3PVA- 2Na Alg (through carbon atom number 10) and Term 1Na Alg- 3PVA- Mid 1Na Alg have the lowest energy gap values^48^. As a result, the influence of Gly addition on the most likely models of blended PVA/ Na Alg polymer is investigated using the final two structures, 3PVA- (C_10_)2Na Alg (for simplicity, 3PVA-2Na Alg) and Term 1 Na Alg − 3PVA- Mid 1 Na Alg. According to the literature, PVA, Na Alg, and glycerin can only form weak hydrogen bonds across the hydroxyl functional groups. Because trimer PVA and dimer Na Alg and glycerin have many OH groups, the contact can take place through one of the OH groups present. Figure 1 depicts the interaction of glycerin model molecules with the 3PVA-2Na Alg model molecule, while Fig. 2 shows the constructed models representing Term 1Na Alg-3PVA-Mid 1Na Alg interacted with different concentrations of glycerin.

### Electronic band gap energy calculation

The electronic band gap energy is an important parameter to consider when studying the reactivity of any electrode material. Because it describes the behavior of electrons when the material is subjected to external changes. As a result, the HOMO/LUMO electronic band gap energy is estimated for all structures under investigation. The variations in the HOMO/LUMO energy of 3PVA-(C_10_)2Na Alg and Term 1Na Alg − 3PVA- Mid 1Na Alg due to glycerin addition are presented in Table 2. According to ref^47^, the E_g_ value of 3PVA-(C_10_)2Na Alg is 0.2908 eV, while that of the structure reflecting the second interaction probability (i.e., Term 1Na Alg − 3PVA- Mid 1Na Alg) is 0.5706 eV.

However, it has been discovered that small variations in the value of the E_g_ value of 3PVA- (C_10_) 2Na Alg have occurred as a result of glycerin addition. When 3PVA - (C_10_) 2Na Alg interacts with 1, 2, 3, 4, and 5 units of glycerin, it becomes 0.302, 0.299, 0.30 8, 0.28 9, and 0.281  eV, respectively. However, there is a valuable idea: after adding 3 glycerin units, the value of E_g_ became less than that of 3PVA- (C_10_) 2Na Alg. The model representing 3PVA- (C_10_) 2Na Alg interacted with 5 units of glycerin is the most likely model for interaction. This means that the probability of interaction increases as the glycerin units increase.

Meanwhile, for the second interaction probability, HOMO/LUMO energy changed to 1.343, 1.34 7, 0.976, 0.607, 0.348, and 0.496  eV for model molecules representing Term 1Na Alg − 3PVA –Mid 1Na Alg- 1Gly, Term 1Na Alg − 3PVA –Mid 1Na Alg- 2Gly, Term 1Na Alg − 3PVA –Mid 1Na Alg- 3Gly, Term 1Na Alg − 3PVA –Mid 1Na Alg- 4Gly, Term 1Na Alg − 3PVA –Mid 1Na Alg- 5Gly, and Term 1Na Alg − 3PVA –Mid 1Na Alg- 6Gly, respectively. The calculated HOMO/LUMO band gap energies are presented in Table 2 for all structures. Additionally, the same behavior for the interaction probability for the first group repeats itself here.

The energy band theory of solid-state physics states that as the band gap narrows, electrode materials’ electronic conductivity will rise. Doping is a common method of reducing the band gap in sodium-ion cathode materials. Cu doping was employed by Jiang et al. to increase the electronic conductivity of the β-NaMnO_2_ layered material. Using the DFT calculation, they discovered that the material’s band gap dropped from 0.7 eV to 0.3 eV due to doping. This suggests that Cu doping enhances the β-NaMnO_2_ material’s electronic conductivity^48^.

**Table 2:** Calculated HOMO/LUMO band gap energy as (eV) at B3LYP/6- 311G(d, p) for 3PVA – 2Na Alg interacted with 1, 2, 3, 4 and 5 units of glycerin and for term 1 na alg − 3PVA –Mid 1 na Alg interacted with 1, 2, 3, 4, 5 and 6 units of glycerin.

First interaction probability		Second interaction probability	
Structure	E_g_	Structure	E_g_
3PVA – (C_10_) 2 Na Alg	0.291^46^	Term 1 Na Alg − 3PVA –Mid 1 Na Alg	0.571^46^
3PVA –(C_10_) 2 Na Alg- 1 Gly	0.302	Term 1 Na Alg − 3PVA –Mid 1 Na Alg- 1 Gly	1.343
3PVA –(C_10_) 2 Na Alg- 2 Gly	0.299	Term 1 Na Alg − 3PVA –Mid 1 Na Alg- 2 Gly	1.347
3PVA –(C_10_) 2 Na Alg- 3 Gly	0.308	Term 1 Na Alg − 3PVA –Mid 1 Na Alg- 3 Gly	0.976
3PVA –(C_10_) 2 Na Alg- 4 Gly	0.289	Term 1 Na Alg − 3PVA –Mid 1 Na Alg- 4 Gly	0.607
3PVA –(C_10_) 2 Na Alg- 5 Gly	0.281	Term 1 Na Alg − 3PVA –Mid 1 Na Alg- 5 Gly	0.348

### Molecular electrostatic potential (MESP)

**Fig. 3:**
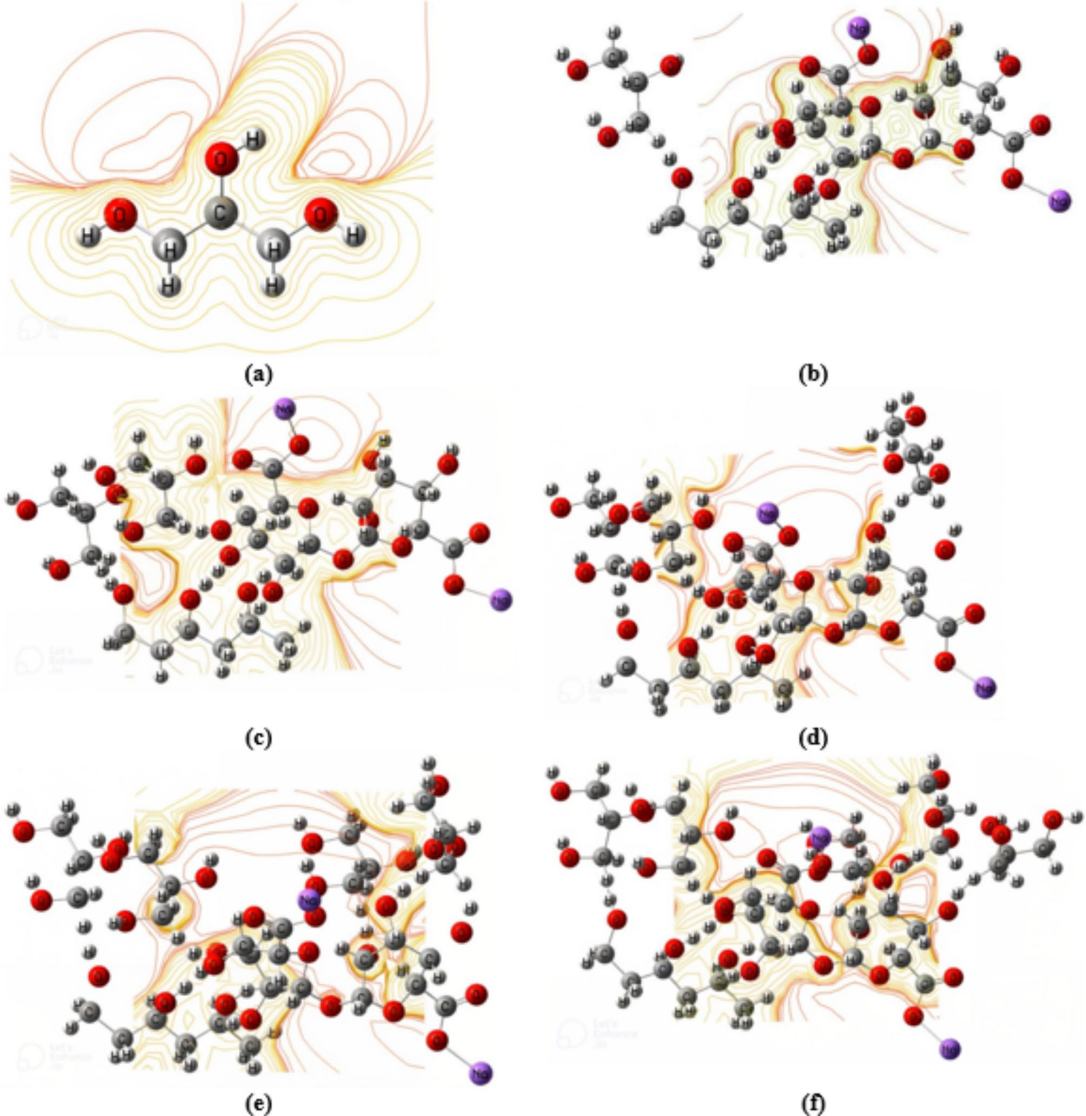
B3LYP/6–311 g(d, p) calculated MESP as contour for : (**a**) Gly and 3PVA − 2Na Alg interacted with (**b**) 1 Gly, (**c**) 2 Gly, (**d**) 3 Gly, (**e**) 4 Gly, and (**f**) 5 Gly.

MESP is defined as the interaction energy between the charge distribution of a molecule and a unit positive charge. MESP is considered an efficient tool to understand and interpret the chemical properties and reactivity. MESP can be used to understand the interaction mechanism between polymeric materials. The MESP depicts the charge distribution within the investigated compounds. Furthermore, MESP offers information regarding the active sites in the materials under investigation^32^. Figure 3 depicts the predicted MESP maps for 3PVA- (C_10_) 2Na Alg, 3PVA-(C_10_) 2Na Alg − 1Gly, 3PVA-(C_10_) 2Na Alg − 2Gly, 3PVA-(C_10_) 2Na Alg − 3Gly, 3PVA-(C_10_) 2Na Alg − 4Gly, and 3PVA-(C_10_) 2Na Alg − 5Gly at the B3LYP/6-311G(d, p) level of theory.

**Fig. 4:**
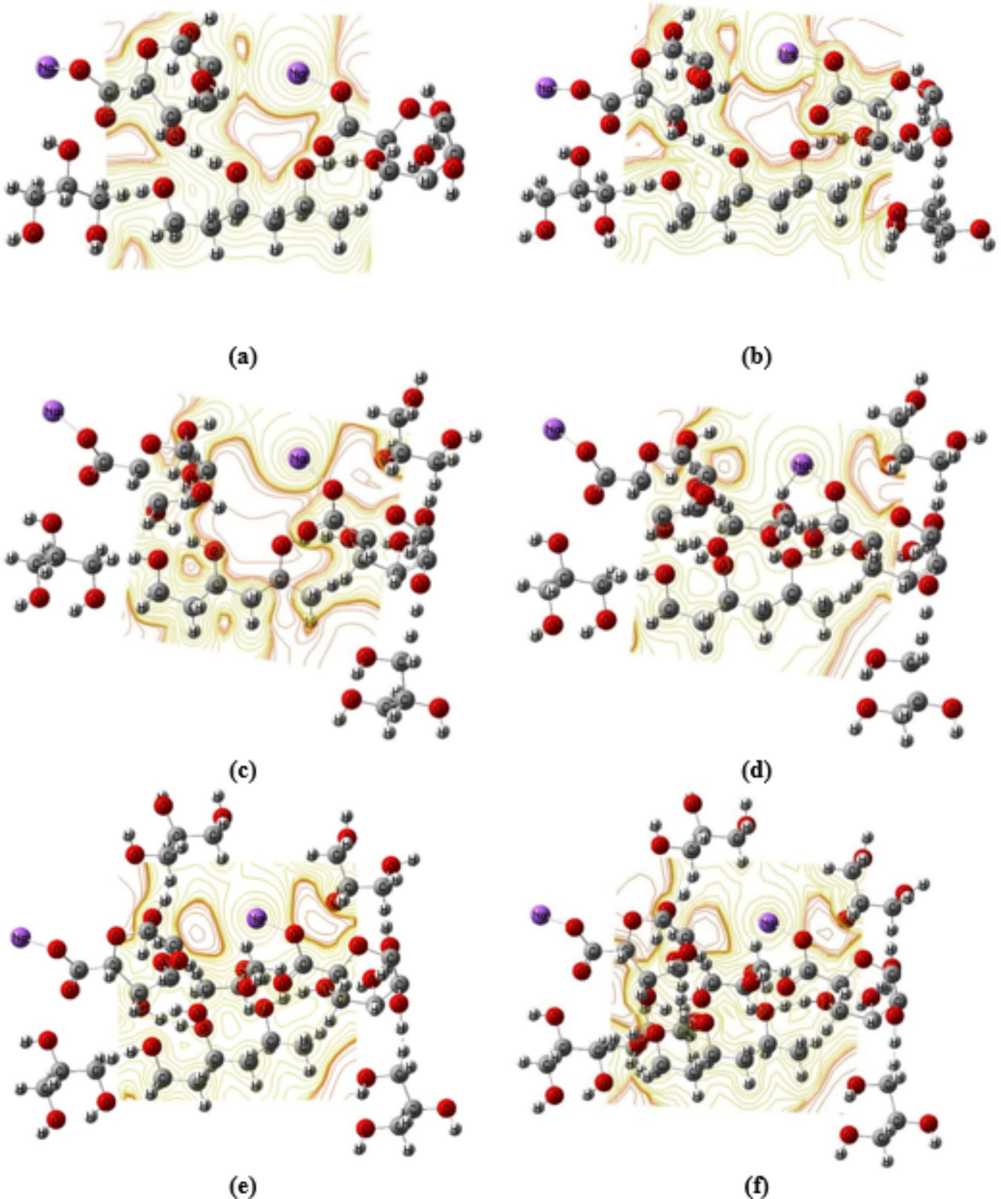
B3LYP/6–311 g(d, p) calculated MESP for Term 1Na Alg- 3PVA –Mid 1Na Alg interacted with (**a**) 1 Gly, (**b**) 2 Gly, (**c**) 3 Gly, (**d**) 4 Gly, (**e**) 5 Gly, and (**f**) 6 Gly.

Meanwhile, Fig. 4 shows the calculated MESP for Term 1Na Alg- 3PVA - Mid 1Na Alg, Term 1Na Alg-3PVA - Mid 1Na Alg- 1Gly, Term 1Na Alg-3PVA - Mid 1Na Alg − 2Gly, Term 1Na Alg-3PVA - Mid 1Na Alg − 3gly, Term 1Na Alg-3PVA - Mid 1Na Alg- 4Gly, Term 1Na Alg- 3PVA - Mid 1Na Alg- 5gly, and Term 1Na Alg-3PVA - Mid 1Na Alg − 6Gly, respectively. The calculated MESP is presented as contour action. This contour is represented by many colors. Each color represents a different electro-negativity value. The red color denotes sites of high electronegativity, or high reactivity. Meanwhile, the yellow color represents the neutral sites within the structures^49–51^. The MESP findings showed that the reactivity of 3PVA- (C_10_) 2Na Alg increased as the red color around the examined models increased. Meanwhile, the red color intensity in the MESP maps of the Term 1Na Alg-3PVA - Mid 1Na Alg model molecule reduced due to their interaction with different glycerin content. The changes in the distribution of the red color around the proposed structures reflect the reactivity, while the increased intensity confirmed the increased electronegativity of 3PVA- (C_10_) 2Na Alg model molecules due to the increasing glycerin content.

### Thermal parameters

Thermal parameters such as enthalpy, entropy, heat capacity, free energy, and heat of formation are calculated for all proposed structures at different temperatures ranging from 200 K to 500 K. In order to describe the behavior of physical systems, it is important to study their thermal behavior as a function of temperature due to their interaction with others, in addition to their electronic behavior, which can be calculated using the equations presented in Table 1. Studying such thermal parameters is considered a significant indicator of the responsibility and stability of such physical systems when they are subjected to different temperatures.

Regarding the enthalpy of trimer PVA, it interacted firstly with dimer Na Alg, throughout the OH group connected to carbon atom number 10, and finally with glycerin. Enthalpy is the measurement of energy in a thermodynamic system. The quantity of enthalpy equals the total content of heat in a system, equivalent to the system’s internal energy plus the product of volume and pressure. In another word, enthalpy tells how much heat and work is added or removed from this substance^52^.

**Fig. 5:**
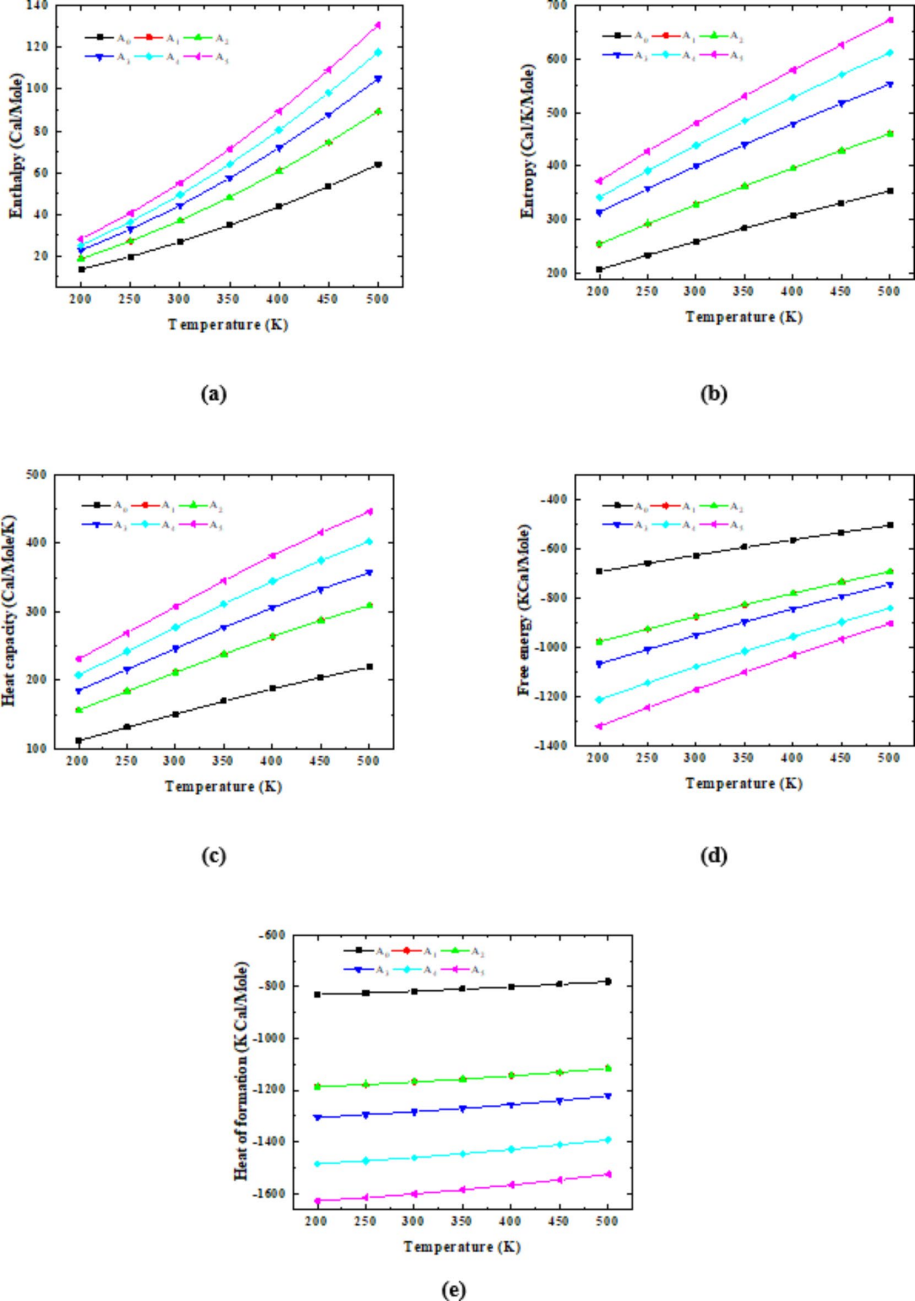
PM6 calculated thermal parameters for pure 3PVA- (C_10_) 2Na Alg (Model A_0_), 3PVA- (C_10_) 2Na Alg − 1 Gly (Model A_1_), 3PVA- (C_10_) 2Na Alg − 2 Gly (Model A_2_), 3PVA- (C_10_) 2Na Alg − 3 Gly (Model A_3_), 3PVA- (C_10_) 2Na Alg − 4 Gly (Model A_4_), and 3PVA- (C_10_) 2Na Alg − 5 Gly (Model A_5_) where, (**a**) enthalpy, (**b**) Entropy, (**c**) Heat capacity, (**d**) Free energy, and (**e**) Heat of formation.

Figure 5 presents the changes that occurred in the enthalpy of 3PVA- (C_10_) 2Na Alg due to the interaction with different concentrations of glycerin. The abbreviations A_0_, A_1_, A_2_, A_3_, A_4_, and A_5_ refer to the model molecules representing 3PVA- (C_10_) 2Na Alg, 3PVA- (C_10_) 2Na Alg − 1 Gly, 3PVA- (C_10_) 2Na Alg − 2Gly, 3PVA- (C_10_) 2Na Alg − 3Gly, 3PVA- (C_10_) 2Na Alg − 4Gly and 3PVA- (C_10_) 2Na Alg − 5Gly. Figure 5a indicated that the enthalpy increased with increasing both the temperature and the glycerin content. Whereas, the enthalpy of the structure representing 3PVA- (C_10_) 2Na Alg − 5Gly (i.e., A5) at 200 K equals 27.966 Cal/Mole, while it equals 13.490 Cal/Mole for 3PVA- 2Na Alg. Finally, this reaction is an endothermic reaction because of the positive value of enthalpy.

Entropy, which is defined as a measure of the unavailable energy in a closed thermodynamic system, is also usually considered a measure of the system’s disorder. Figure 5b represents the dependence of the entropy of 3PVA- (C_10_)2Na Alg upon temperature and how it interacted with different units of glycerin. The figure shows a linear behavior with increasing temperature from 200 K up to 500 K. It is clear from Fig. 5b that the entropy of the 3PVA- (C_10_)2Na Alg model tends to 200 Cal/K/Mole at 200 K since 3PVA- (C_10_)2Na Alg model exhibits less lattice disorder. As temperature increases, 3PVA- (C_10_)2Na Alg model becomes disorderly and explains why the entropy increases with temperature. Also, it is clear that the structure of 3PVA-C_10_ 2Na Alg- 5 Gly possesses the highest value of entropy.

The same behavior is observed in Fig. 5c, which represents the variation of heat capacity with temperature. The heat capacity is the amount of heat required to change the temperature of a given amount of matter by 1 °C^47^. Figure 5c presents the changes that occurred in the heat capacity of the 3PVA- (C_10_) 2Na Alg model molecule due to the interaction with 1, 2, 3, 4, and 5 units of glycerin. The figure shows that the heat capacity of the 3PVA- (C_10_) 2Na Alg model increases linearly with temperature. The observed increase in the heat capacity with temperature is attributed to the phonon thermal vibrations. Also, it is evidence that increasing glycerin content causes the heat capacity of the 3PVA- (C_10_)2Na Alg model to increase. Additionally, the structure presents 3PVA- (C_10_) 2Na Alg − 5Gly has the highest heat capacity value in contrast to the other structures.

Other parameters, such as free energy and final heat of formation, are calculated for the studied structures, which are presented in Fig. 5d and e, respectively. The final heat of formation is the amount of heat released or absorbed during the formation of a certain pure substance from its constituent elements at constant pressure. Free energy could be defined as the energy-like property, meaning that its magnitude depends on the amount of a substance in each thermodynamic state. The values of free energy and heat of formation of the 3PVA- (C_10_) 2Na Alg − 5Gly are the lowest values and equal − 1,318.338 and − 1,628.154 Kcal/mol, respectively. In contrast, the free energy and heat of formation values for the structure representing 3PVA- (C_10_) 2Na Alg are the highest in comparison to others, equalling − 690.340 and − 830.673 Kcal/mol, respectively. As seen in Fig. 5, the various thermal characteristics alter as a result of interaction with glycerin. The negative values of the Gibbs free energy indicate that the supposed structures are stable.

**Fig. 6:**
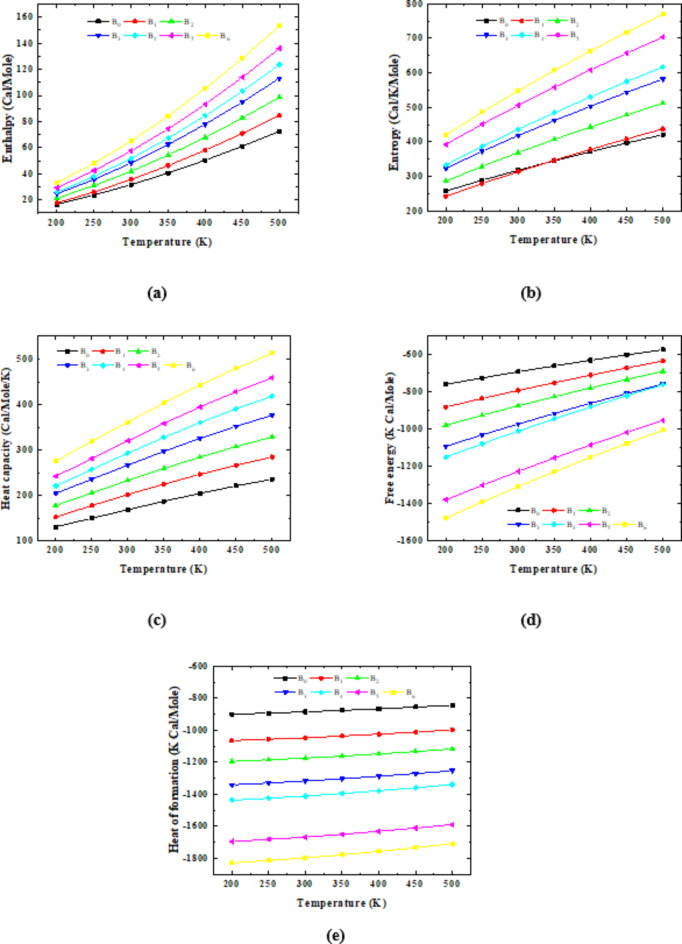
PM6 calculated thermal parameters for pure Term 1 Na Alg- 3PVA- Mid 1 Na Alg (Model B_0_), Term 1 Na Alg- 3PVA- Mid 1 Na Alg – 1 Gly (Model B_1_), Term 1 Na Alg- 3PVA- Mid 1 Na Alg – 2 Gly (Model B_2_), Term 1 Na Alg- 3PVA- Mid 1 Na Alg – 3 Gly (Model B_3_), Term 1 Na Alg- 3PVA- Mid 1 Na Alg – 4 Gly (Model B_4_), Term 1 Na Alg- 3PVA- Mid 1 Na Alg – 5 Gly (Model B_5_), and Term 1 Na Alg- 3PVA- Mid 1 Na Alg – 6 Gly (Model B_6_) where, (**a**) enthalpy, (**b**) Entropy, (**c**) Heat capacity, (**d**) Free energy, and (**e**) Heat of formation.

The second mode of interaction between trimer PVA and dimer Na Alg, on the other hand, occurs throughout the terminal and middle OH groups present in the trimer PVA structure. Thermal parameters, like the first group, are calculated at the same level of theory. Figure 6a–e shows the fluctuation in enthalpy, entropy, heat capacity, free energy, and final heat of formation. Figure 6a–c indicates that the enthalpy, entropy, and heat capacity of Term 1 Na Alg- 3PVA- Mid 1 Na Alg exhibit the same behavior as the first group when interacting with 1, 2, 3, 4, 5, and 6 units of glycerin. Additionally, their values increased gradually with increasing temperature. Also, the values of enthalpy, entropy, and heat capacity increased with increasing the glycerin content in the proposed model of Term 1 Na Alg − 3PVA- Mid 1 Na Alg. The abbreviations B_0_, B_1_, B_2_, B_3_, B_4_, B_5_, and B_6_ refer to the structures representing: Term 1 Na Alg − 3PVA- Mid 1 Na Alg, Term 1 Na Alg- 3PVA- Mid 1 Na Alg − 1 Gly, Term 1 Na Alg- 3PVA- Mid 1 Na Alg − 2gly, Term 1 Na Alg- 3PVA- Mid 1 Na Alg − 3gly, Term 1 Na Alg- 3PVA- Mid 1 Na Alg − 4 Gly, Term 1 Na Alg- 3PVA- Mid 1 Na Alg − 5 Gly, and Term 1 Na Alg- 3PVA- Mid 1 Na Alg − 6 Gly, respectively. As presented in Fig. 6a–c, it is obvious that the values of enthalpy, entropy, and heat capacity increased with increasing glycerin units from 1 to 6 units.

Also, the structure representing Term 1 Na Alg- 3PVA- Mid 1 Na Alg- 6 Gly possesses the highest enthalpy, entropy, and heat capacity values in comparison with others. Where, their values increased from 16.703 Cal/Mole, 257.990 Cal/Mole/K, and 131.323 KCal/Mole for Term 1 Na Alg − 3PVA- Mid 1 Na Alg, to 33.223 Cal/Mole, 420.038 Cal/Mole/K, and 275.923KCal/Mole for Term 1 Na Alg − 3PVA- Mid 1 Na Alg − 6 Gly, respectively.

However, Figs. 6d and e present the temperature dependence of free energy and the final heat of formation (HF). HF can be defined as the change in enthalpy that occurs during the formation of one mole of a substance from its elements in their natural and standard states. From the figure, the free energy and final heat of formation for all studied structures show a linear dependence on temperature, where they increased linearly and gradually with increasing temperature. Also, the figures confirmed that the structure representing Term 1 Na Alg − 3PVA- Mid 1 Na Alg − 6 Gly has the lowest free energy and the lowest HF. Both parameters decreased from − 758.337 to − 899.741 K Cal/Mole to -1,476.591 and − 1,828.523 K Cal/Mole for Term 1 Na Alg − 3PVA- Mid 1 Na Alg − 6 Gly. As noted from the results, HF decreased with increasing the units of glycerin. This means that the interaction is acquired with less energy due to the increasing number of functional groups that increase the reactivity. This confirmed that the plasticized PVA/Na Alg can be used in rechargeable batteries due to its high reactivity.

In general, there are two types of temperature impacts: effects at low and high temperatures. The majority of the effects of low temperatures occur in high-latitude countries like Greenland Island, Canada, and Russia. The wintertime outdoor temperatures in these places are significantly below zero °C. The life and performance of LIBs will be impacted by such low temperatures, particularly those used in plug-in hybrid electric vehicles, pure electric vehicles, and hybrid electric vehicles. Space travel is another frigid environment where LIBs are used. The temperature on Mars can drop to as low as -120 °C, for instance, which presents a significant obstacle to the use of LIBs in astrovehicles for space travel. Low operating temperatures cause LIBs to exhibit slow charge-transfer velocity and chemical reaction activity, which in turn causes a decrease in lithium-ion diffusivity within the electrodes and ionic conductivity in the electrolytes. This decline will lead to a decrease in energy and power capacity and occasionally even a breakdown in performance^53^.

The high temperature effects occur in a much wider range of application environments, including both high and low temperature environments, in contrast to the low temperature effects, which are primarily restricted to low temperature application environments. Low temperature effects are largely determined by the ambient temperature, whereas high temperature effects are typically more accurately attributed to the high internal temperature of LIBs during operation.

Heat generation within LIBs occurs at high current states, including fast charging and fast discharging rates, and is the cause of the high internal temperature. The effects of high temperatures will also cause the batteries’ performance to deteriorate, including capacity and power loss. Generally speaking, loss of lithium and reduction of active materials at high temperatures will cause capacity loss, whereas loss of power is caused by an increase in internal resistance. Thermal runaway will occur if the temperature gets out of control, which could cause self-ignition or even an explosion in certain situations.

### QSAR parameters

QSAR calculations are computational or mathematical modeling methods to reveal the relationships between biological activities and the structural properties of chemical compounds. All constructed molecules are optimized, and some QSAR properties are calculated at the PM6 level. Table 3 presents some of the calculated QSAR descriptors. Charge, TDM, total energy (E), ionization potential (IP), Log P, and Polarizability are examples of such descriptors (see Table 1 for the equations that determine IP and Log P).

**Table 3:** Some of the QSAR descriptors such as total charge, total dipole moment (TDM), total energy (E), ionization potential (IP), log P and polarizability calculated at the PM6 level of the semi-empirical method.

Structure	Charge (coulomb)	TDM (debye)	E (eV)	IP (eV)	Log *p*	Polarizability (Å)
Gly	0	2.788	− 141.833	− 5.157	− 1.081	4.696
3PVA-2 Na Alg	0	6.840	− 200092.503	− 9.341	− 3.100	29.690
3PVA − 2 Na Alg- 1 Gly	0	17.990	− 996.837	− 9.256	− 3.537	35.076
3PVA − 2 Na Alg- 2 Gly	0	8.848	− 1108.440	− 9.393	− 5.261	40.665
3PVA − 2 Na Alg- 3 Gly	0	5.874	− 1238.740	− 9.393	− 6.342	45.177
3PVA − 2 Na Alg- 4 Gly	0	7.568	− 1372.075	− 9.248	− 7.423	50.239
3PVA − 2 Na Alg- 5 Gly	0	12.779	− 1548.031	− 9.323	− 8.504	54.638

As a consequence of the computations, all of the investigated structures have a zero total charge because they are in the ground state. For the first interaction probability, the TDM of 2.788 Debye for glycerin and 6.840 Debye for 3PVA-(C_10_) 2Na Alg is enhanced to 17.990, 8.848, 5.874, 7.568, and 12.779 Debye for 3 PVA-(C_10_) 2Na Alg interacted with 1, 2, 3, 4, and 5 units of glycerin, respectively. The higher the TDM value, the greater the reactivity with the surrounding media.

Total energy (E) is also calculated, and it is found that for glycerin and 3PVA-(C_10_)2 Na Alg, the E values equal − 141.833 eV and − 200092.503 eV. Meanwhile, the structure representing 3PVA- (C_10_)2 Na Alg interacted with 1, 2, 3, 4, and 5 units of glycerin; E became − 996.837, -1108.440, -1238.740, -1372.075, and − 1548.031 eV, respectively. Increasing the glycerin content causes the total energy to decrease, and hence the reactivity increased. Depending on the calculation of total energy, it is concluded that the model molecule representing 3PVA- 2Na Alg- 5 Gly is more reactive than other model molecules. This phenomenon is related to its structure, where 3PVA-(C_10_) 2 Na Alg possess only two –COONa groups, while the other structures possess two –COONa groups with several OH groups, which means that their reactivity for the surrounding medium increased.

Additionally, IP is also included in this study for all structures. IP becomes a very important indicator for the reactivity of the studied models. The quantity of energy required to move an electron from a point in the molecule to infinity is called the ionization potential. This represents how the molecule becomes ionized (i.e., reactive). The higher the ionization potential, the lower the reactivity. The results of IP were − 9.256, -9.393, -9.393, -9.248 and − 9.323 eV for 3PVA-(C_10_) 2 Na Alg interacted with 1, 2, 3, 4, and 5 units of glycerin, respectively, instead of -5.157 and − 9.341 eV for glycerin and 3PVA-(C_10_) 2Na Alg, respectively. As the IP values decreased due to glycerin addition, the molecules reactivity increased, which increased the applicability of using PVA/Na Alg/glycerin model molecules in electrochemical devices.

The fifth descriptor presented in Table 3 is Log P, which is the partition coefficient logarithm that describes whether the studied structure is hydrophilic or hydrophobic. The negative value of Log P indicates a hydrophilic molecule, which means that it is soluble in water and less soluble in organic solvents. However, the positive value refers to the inverse process.

Based on the obtained results, it is concluded that all structures are hydrophilic as they have Log P values of -3.537, -5.261, -6.342, -7.423, and − 8.504 for 3PVA-(C_10_) 2Na Alg − 1Gly, 3PVA- (C_10_) 2Na Alg − 2Gly, 3PVA-(C_10_) 2Na Alg − 3Gly, 3PVA-(C_10_) 2Na Alg − 4Gly and 3PVA-(C_10_) 2Na Alg − 5Gly, respectively, instead of -1.081 for glycerin and − 3.100 for 3PVA-(C_10_) 2Na Alg. This means that the properties of the studied structures will change with the incorporation of water molecules into their structures.

Finally, the polarizability of all structures is also calculated at the PM6 level of the semi-empirical method. Previously, it was stated that the polarizability of most materials depends on different factors. The most important factor is the volume of the studied structures. For all structures supposed in the first way of interaction between 3PVA and 2 Na Alg (interaction proceeds through carbon atom number 10), the polarizability increased due to glycerin addition. The polarizability increased from 29.690 Å to 35.076, 40.665, 45.177, 50.239, and 54.638 Å due to the interaction with 1, 2, 3, 4, and 5 units of glycerin. So, it is found that the largest polarizability belongs to the 3PVA-(C_10_) 2Na Alg − 5Gly model molecules, while the smallest one is that of 3PVA-(C_10_) 2Na Alg which equals 29.690 Å.

The assessment of the QSAR descriptors leads to the conclusion that the structure representing 3PVA-(C_10_) 2Na Alg − 5Gly has the maximum reactivity for the first proposed interaction.

Regarding the second way of interaction between trimer PVA and dimer Na Alg, the results indicated that their charges were similar to those of the first interaction proposed in the last section. Where the electronic charges equal zero for all the structures, which means that they are all in their ground state.

As presented in Table 4, the TDM value of Term 1 Na Alg − 3PVA- Mid 1 Na Alg (calculated at the PM6 level) increased from 11.581 Debye to 15.756, 19.720, 21.756, 22.732, 15.507, and 15.756 for Term 1 Na Alg − 3PVA- Mid 1 Na Alg interacted with 1, 2, 3, 4, 5, and 6 units of glycerin, respectively. However, the total energy decreased with increasing the number of glycerin units and became − 996.985, -1129.013, -1267.211, -1321.775, -1418.964, and − 1637.432 eV when Term 1 Na Alg − 3PVA- Mid 1 Na Alg interact with a number of glycerin units, starting from 1 to 6 units, respectively.

**Table 4:** Some of the QSAR descriptors such as total charge, total dipole moment (TDM), total energy (E), ionization potential (IP), log P, polarizability calculated at PM6 level of the semi-empirical quantum mechanical calculations.

Structure	Total charge (coulomb)	TDM (debye)	E (eV)	IP (eV)	Log *p*	Polarizability (Å)
Term 1 Na Alg − 3PVA –Mid 1 Na Alg	0	11.581	− 884.895	− 9.385	− 3.643	31.703
Term 1 Na Alg − 3PVA –Mid 1 Na Alg- 1 Gly	0	15.756	− 996.985	− 8.946	− 5.334	39.958
Term 1 Na Alg − 3PVA –Mid 1 Na Alg- 2 Gly	0	19.720	− 1129.013	− 8.848	− 6.415	44.403
Term 1 Na Alg − 3PVA –Mid 1 Na Alg- 3 Gly	0	21.756	− 1267.211	− 8.430	− 7.496	49.850
Term 1 Na Alg − 3PVA –Mid 1 Na Alg- 4 Gly	0	22.732	− 1321.775	− 9.537	− 9.096	52.121
Term 1 Na Alg − 3PVA –Mid 1 Na Alg- 5 Gly	0	15.507	− 1418.964	− 7.997	− 9.861	56.170
Term 1 Na Alg − 3PVA –Mid 1 Na Alg- 6 Gly	0	15.756	− 1637.432	− 8.900	− 10.535	63.198

For the second interaction probability, IP, Log P, and polarizability are also calculated at the PM6 level of theory. As a result, they considered three of the most powerful descriptors of the molecule’s reactivity. IP increased from − 9.385 eV to -8.946, -8.848, -8.430, -9.537, -7.997, and − 8.900 eV for the structure representing Term 1 Na Alg- 3PVA- Mid 1Na Alg interacted with 1, 2, 3, 4, 5, and 6 glycerin units, respectively. However, the computed values of Log P are found to be lower as a result of Term 1 Na Alg − 3PVA- Mid 1 Na Alg plasticization with glycerin. Whereas increasing glycerin from 1 to 6 results in values of -5.334, -6.415, -7.496, -9.096, -9.861, and − 10.53 instead of -3.643. Finally, the polarizability data demonstrate that increasing the glycerin content causes the polarizability of Term 1 Na Alg- 3PVA- Mid 1 Na Alg to increase. Whereas, the polarizability of the Term 1 Na Alg- 3PVA- Mid 1 Na Alg model molecule increased from 31.703 Å to 63.198 Å upon the interaction with 6 glycerin units. Note that increasing the number of glycerin units in the second probability of the interaction is to confirm that despite the huge number of atoms and the complexation of the structures, the properties are still enhanced with increasing the glycerin content. Therefore, it may be said that affordable PVA/Na Alg/glycerin models can partially replace lithium-ion batteries, but more research and development are needed.

### Quantum theory of atoms in molecules (QTAIM) analysis

**Fig. 7:**
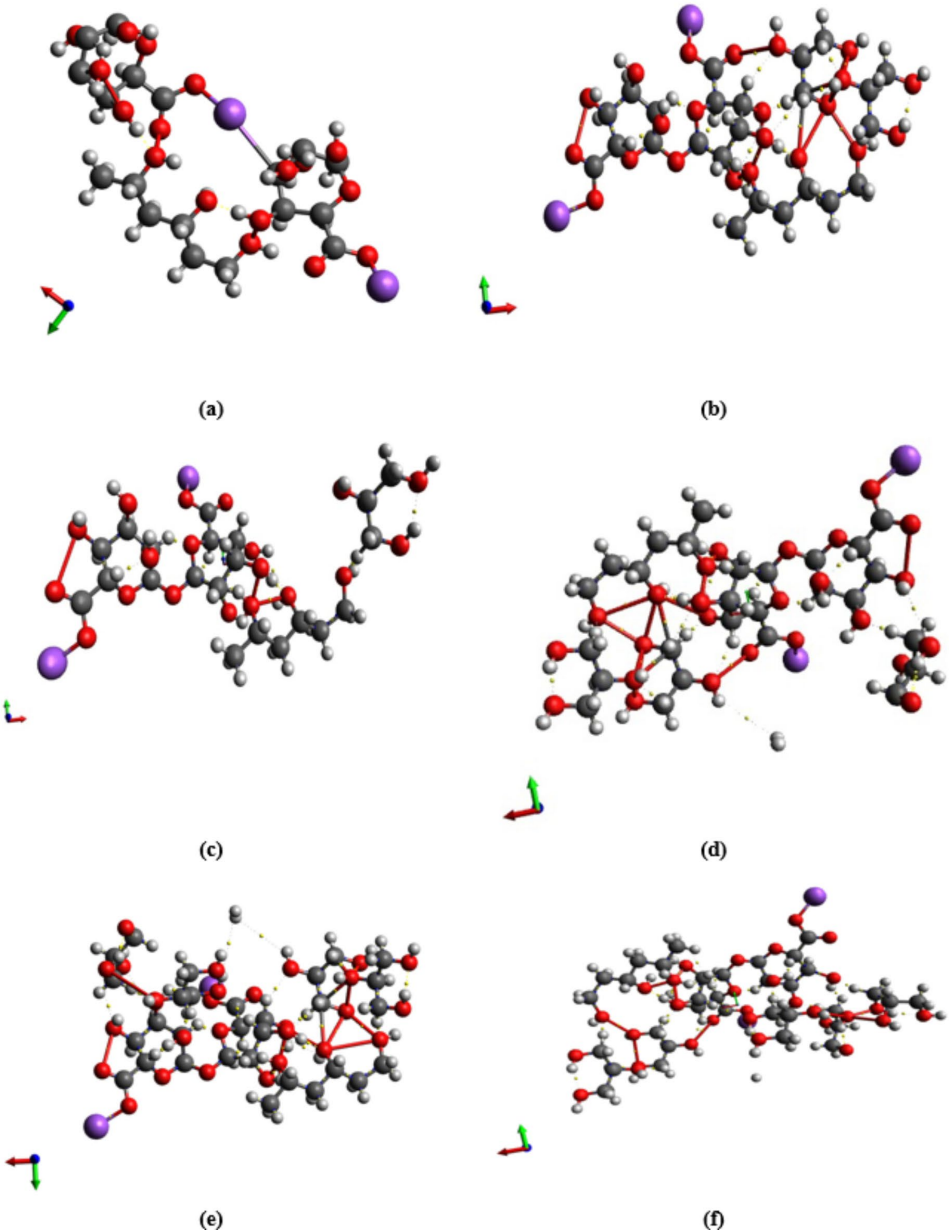
QTAIM analyses for 3PVA − 2Na Alg model molecule interacted with (**a**) 0 Gly, (**b**) 1 Gly, (**c**) 2 Gly, (**d**) 3 Gly, (**e**) 4 Gly, and (**f**) 5Gly.

**Fig. 8:**
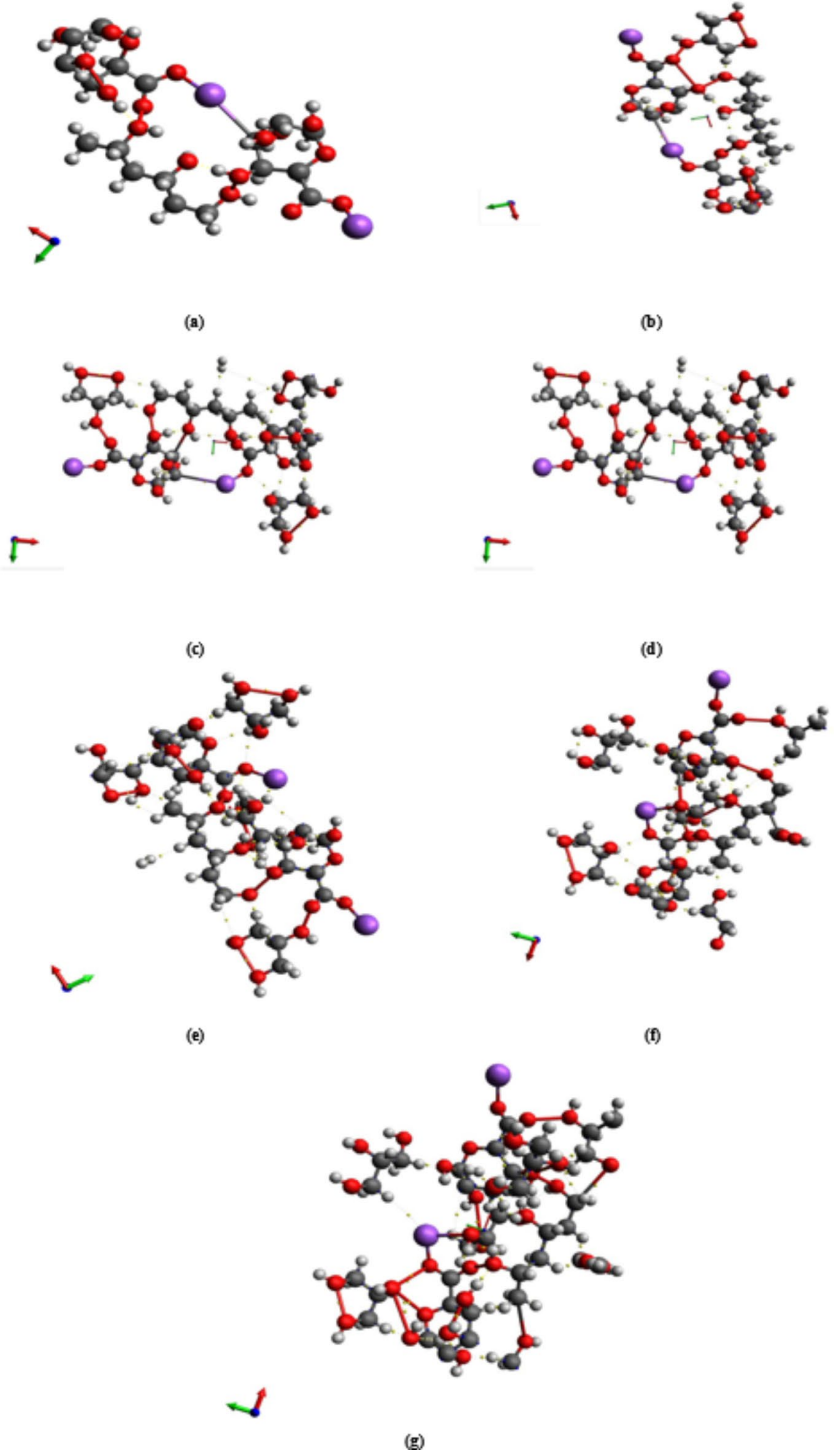
QTAIM analyses for Term 1Na Alg- 3PVA –Mid 1Na Alg model molecule interacted with (**a**) 0 Gly, (**b**) 1 Gly, (**c**) 2 Gly, (**d**) 3 Gly, (**e**) 4 Gly, (**f**) 5Gly, and (**g**) 6 Gly.

Characterizing the binding capacity of the surface with the adsorbate and estimating the unique interactions among the systems require knowledge of the types of bonding that exist between any two atoms, the intricacies of the inter- and intra-molecular interactions, and the electron density distribution of the surfaces and adsorbents. The electron density at bond critical points (BCPs) between interacting atoms is crucial for assessing the bonding interactions’ strength in QTAIM analysis. Stronger electronic charge densities, which correlate to more stable and covalent interactions, are typically indicated by higher electron density values at these critical points. In addition, a covalent (shared) interaction is suggested if both total electron energy density (H(r)) and Laplacian charge density (∇2ρ(r)) are less than 0. On the other hand, non-covalent (closed-shell) interactions like weak hydrogen bonds, van der Waals forces, and electrostatic interactions are indicated when ∇2ρ(r) and H(r) are greater than 0^54^. QTAIM analysis reveals the nature of non-covalent interactions within the studied structures, as shown in Figs. 7 and 8. Based on the analysis, model molecules representing 3PVA − 2Na Alg and Term 1 Na Alg − 3PVA –Mid 1 Na Alg exhibit greater stability than those interacted with different units of glycine. This is because several non-covalent interactions, which are more prevalent in the sodium alginate structure, such as electrostatic interactions and hydrogen bonding, enable sodium alginate to stabilize the composite. Furthermore, our findings demonstrate the importance of the non-covalent interactions between the 3PVA − 2Na Alg and Term 1 Na Alg − 3PVA –Mid 1 Na Alg model molecules and glycine, suggesting that glycine plays a major role in modifying the composite’s overall electronic environment.

### Cell voltage of the PVA/Na Alg/Glycerin models

The Gibbs free energy of the reaction is equal to the internal energy change before and after the reaction for the cell system with constant volume at low temperature and normal pressure. Based on this, the internal energy change of the reaction can be calculated to determine the mass/volume energy density of the battery as well as the specific capacity of the electrode materials in the battery. By calculating the Gibbs free energy released by the entire electrochemical system during the transfer of unit electrons, we can determine the cell voltage of the electrode. We can also determine the stability of the electrode structure by calculating the cohesive energy, free energy, formation energy, etc^55^.

The cell voltage (V_cell_) was calculated using the Nernst equation V_cell_ = - ΔG_cell_/ZF. Where ΔD G_cell_, F, and Z are the change in Gibbs free energy, Faraday constant (96500 C/mol), and the Na^+^ charge, respectively.

In the DFT calculation at 0 K, ΔG_cell_ can be written as:

7 $$\Delta {\text{G}}_{{{\text{cell}}}} {\mkern 1mu} = {\mkern 1mu} \Delta {\text{E}}_{{{\text{cell}}}} + {\text{ P}}\Delta {\text{V}}_{{{\text{cell}}}} - {\text{T}}\Delta {\text{S}}_{{{\text{cell}}}} .$$

It has previously been shown that the volume changes and entropy contributions in Eq. (7) to the cell voltage calculation are insignificant. Their impact is less than 0.01 V on the cell voltage^56^. As a result, the **Δ**E_cell_ and **Δ**G_cell_ are equal when the terms PΔV_cell_ and TΔS_cell_ are removed from Eq. (7). The calculated cell voltage for the PVA/Na Alg based glycerin electrolytes are presented in Table 5. Table 5 presents the cell voltage of different electrode materials reported in the literature. To fully utilize the high-voltage benefits of LiNiPO_4_, the operating voltage must be lowered because the high-voltage electrolyte that has been developed can only stay relatively stable in the range of less than 4.8 V. Accordingly, it is concluded that plasticized PVA/Na Alg is a good option for rechargeable batteries and can partially replace lithium-ion batteries due to their multifunctionality, but requires further improvement and investigations.

**Table 5:** Cell voltage of PVA/Na Alg model molecules interacted with different units of glycerin.

Structure	Cell voltage (V)
3PVA –(C_10_) 2 Na Alg- 1 Gly	2.488
3PVA –(C_10_) 2 Na Alg- 2 Gly	0.103
3PVA –(C_10_) 2 Na Alg- 3 Gly	0.786
3PVA –(C_10_) 2 Na Alg- 4 Gly	1.323
3PVA –(C_10_) 2 Na Alg- 5 Gly	0.958
Term 1 Na Alg − 3PVA –Mid 1 Na Alg- 1 Gly	1.037
Term 1 Na Alg − 3PVA –Mid 1 Na Alg- 2 Gly	0.856
Term 1 Na Alg − 3PVA –Mid 1 Na Alg- 3 Gly	1.011
Term 1 Na Alg − 3PVA –Mid 1 Na Alg- 4 Gly	0.397
Term 1 Na Alg − 3PVA –Mid 1 Na Alg- 5 Gly	2.243
Term 1 Na Alg − 3PVA –Mid 1 Na Alg- 6 Gly	0.843
LiNiPO_4_	5.100^37^
LiNiPO_4_/Ti	4.210^37^
LiNiPO_4_/Nb	3.760^37^
LiNiPO_4_/Ta	3.500^37^
Graphyne-like BN layer (BN-yne)	2.100 ^5^
LiFeSO_4_F	3.600 ^57^

Finally, in order to increase the working voltage of electrode materials for rechargeable batteries, the band gap energy should be reduced^58^. As presented in Table 2, the energy gap of PVA/Na Alg models was reduced due to the interaction with different units of glycerin. This means that the lowest unoccupied molecular orbital (LUMO) energy of PVA/Na Alg models is typically reduced by the addition of an electron-withdrawing group. Additionally, in order to improve electrical conductivity, the π-conjugation structure is frequently extended to close the energy gap between the LUMO and the highest occupied molecular orbital (HOMO). According to the molecular orbital theory, LUMO energy is used to attract electrons, whereas HOMO energy indicates the capacity to release electrons. Moreover, the electron-releasing/obtaining during the charge/discharge process can be understood as the intrinsic nature of the electrochemical behavior of the organic cathode materials for NIBs. The electrochemical performance of organic materials is correlated with their molecular orbitals (HOMO/LUMO energy and gap). As a result, understanding and applying this relationship can advance the development of high-performance batteries.

## Conclusion

In this work, the electronic, thermal, and QSAR descriptors of plasticized PVA/Na Alg were investigated using the first principle calculations. The electronic structure of materials, including the band gap and MESP, can be accurately predicted through DFT calculations at the B3LYP/6-311G(d, p) model, while thermal parameters and QSAR descriptors were calculated at the PM6 level. It can therefore predict the physical and chemical characteristics of different molecules that make up the electrode materials of sodium-ion batteries, significantly speeding up the process of material identification, optimization, and characterization. The interaction between glycerin and PVA/Na Alg resulted in more reactive structures as the E_g_ decreased to 0.2814 eV and the TDM increased to 22.732 Debye. According to the MESP maps, the electron density distribution around PVA/Na Alg models changed significantly as a result of its interaction with glycerin. Furthermore, the MESP maps illustrated the distribution of the electrostatic potential in the whole molecule, aiding in predicting the molecule’s reactivity. Furthermore, enthalpy of the PVA/Na Alg model molecule (first interaction mechanism) increased from 26.608 to 54.869 Cal/Mole, entropy increased from 260.296 to 480.508 Cal/K/Mole, heat capacity increased from 150.494 to 307.325 Cal/Mole/K, and free energy increased from − 622.853 to -1168.94 K/Cal/Mole with increasing glycerin content to five units. Additionally, the results demonstrated that the reactivity of the PVA/Na Alg structures improved with glycerin interaction. Also, it has been observed that increasing the glycerin content increases the total energy for the first probability of the interaction, thus increasing polarizability, while decreasing the heat of formation and ionization potential. Additionally, it is found that all structures are hydrophilic. Finally, the cell voltage decreased to 2.488 V due to glycerin addition in comparison with that of LiNiPO_4_ reported in the literature. The findings of this work have improved a theoretical idea for the design of new high-voltage materials. As a result, it is possible to infer that quantum mechanical simulations are a useful tool for understanding the mechanism of interaction of the examined polymer matrix, which is an important step towards investigating the electronic characteristics of polymeric materials as they play a critical role in the development of novel materials for a variety of applications.

## Data Availability

The data will be available upon request. Contact Medhat A. Ibrahim, Email: ma.khalek@nrc.sci.eg.
